# Sudden Cardiac Death Due to Ventricular Arrhythmia in Acute Coronary Occlusion: Potential Roles of the Sinoatrial Nodal Artery and Conus Artery

**DOI:** 10.3390/jcdd12060210

**Published:** 2025-05-31

**Authors:** Justine Bhar-Amato, Aurojit Roy, Benjamin Lambert, Sofia Kassou, Stephen P. Hoole, Sharad Agarwal

**Affiliations:** Department of Cardiology, Royal Papworth Hospital, Papworth Road, Trumpington, Cambridge CB2 0AY, UK; justine.bhar.amato@gmail.com (J.B.-A.);

**Keywords:** sudden cardiac death, acute myocardial infarction, ventricular arrhythmia, sinoatrial nodal artery, conus artery, non-LAD culprit, long term outcome

## Abstract

Background: Despite advances in the management of coronary disease, the incidence of sudden cardiac death (SCD) in the context of coronary artery disease (CAD) remains significant and unpredictable. We investigated the patient and angiographic characteristics, and predictors of long-term outcomes, of patients presenting with cardiac arrest in the context of acute coronary occlusion, to elucidate possible mechanisms of ventricular arrhythmia. Methods: A total of 127 consecutive patients presenting to a tertiary cardiac centre with pulseless ventricular tachycardia or ventricular fibrillation during acute myocardial infarction (AMI) were compared to 100 patients with uncomplicated AMI (Cohort A). We also compared a separate group comprising 20 patients with acute RCA occlusion complicated by cardiac arrest and 24 patients with uncomplicated inferior STEMI (Cohort B). Results: In Cohort A, there was a higher incidence of proximal lesions in the arrest group (55% vs. 41%, *p* < 0.05). There was an almost equivalent incidence of both LAD and non-LAD (RCA/Cx) infarcts presenting with cardiac arrest. In the non-LAD arrest patients, sinoatrial nodal artery (SANa) involvement was seen in 77%, compared with 33% in non-arrest patients (*p* < 0.005). In Cohort B, involvement of the SANa or conus artery (CA) was found in 74% of arrest versus 21% of non-arrest patients (*p* < 0.005). Cohort A patients were followed up for 3.8 to 8.7 years, and at the end of this period, 22% of arrest patients and 16% of non-arrest patients were deceased. Mortality <30 days was highest in the arrest group (43% vs. 7%, *p* < 0.05). Beyond 30 days, there were no differences in all-cause mortality between arrest and non-arrest patients. There were more cardiac causes of death in the arrest group (54% vs. 20%, *p* < 0.05). Conclusions: VT/VF arrest due to acute coronary occlusion was more common in those with proximal disease and there was an increased incidence of SANa and/or CA involvement in non-LAD infarcts. Short-term mortality was higher in patients with cardiac arrest post-AMI, but beyond 30 days there was no significant difference.

## 1. Introduction

Sudden cardiac death (SCD) is an unexpected death, presumed due to a cardiac cause, occurring within 1 h of the onset of symptoms, if witnessed, or within 24 h of the individual last being seen alive and healthy [1,2]. The estimated global incidence of SCD is 50–100 per 100,000, and as much as 80% of cases are estimated as attributable to coronary artery disease (CAD); cardiac arrest is not infrequently the initial manifestation of CAD [3]. It can occur with myocardial ischaemia, acute myocardial infarction (AMI) or in the setting of chronic coronary disease.

The acute post-infarct period is characterised by dynamic ischaemia and reperfusion that alters the electrophysiological properties of myocardium, rendering it capable of triggering and sustaining ventricular arrhythmia (VA).

VA in the context of acute infarcts is most often associated with left anterior descending artery (LAD) occlusion. There are, however, case reports of non-dominant right coronary artery (RCA) occlusion being associated with VA [4,5]. Bradycardia-induced VA is a recognised phenomenon and branches arising from the RCA or Circumflex (Cx) supply proximal portions of the cardiac conduction system, including the sinoatrial node (SAN) and atrioventricular node (AVN) [4,6]. Another potential trigger is the conus artery (CA), most commonly arising from the proximal RCA and supplying the arrhythmogenic right ventricular outflow tract, as evidenced by inadvertent cannulation or occlusion being almost invariably associated with VA [4,7,8].

We propose that the SAN artery and CA are two potential contributors to the VA mechanism in non-LAD AMI. We sought to investigate the characteristics of patients admitted to a tertiary cardiac centre with acute ST-elevation myocardial infarction (STEMI) associated with cardiac arrest due to VA and investigated whether the sinoatrial nodal artery (SANa) and CA was involved in non-LAD infarcts.

## 2. Methods

**Figure 1:**
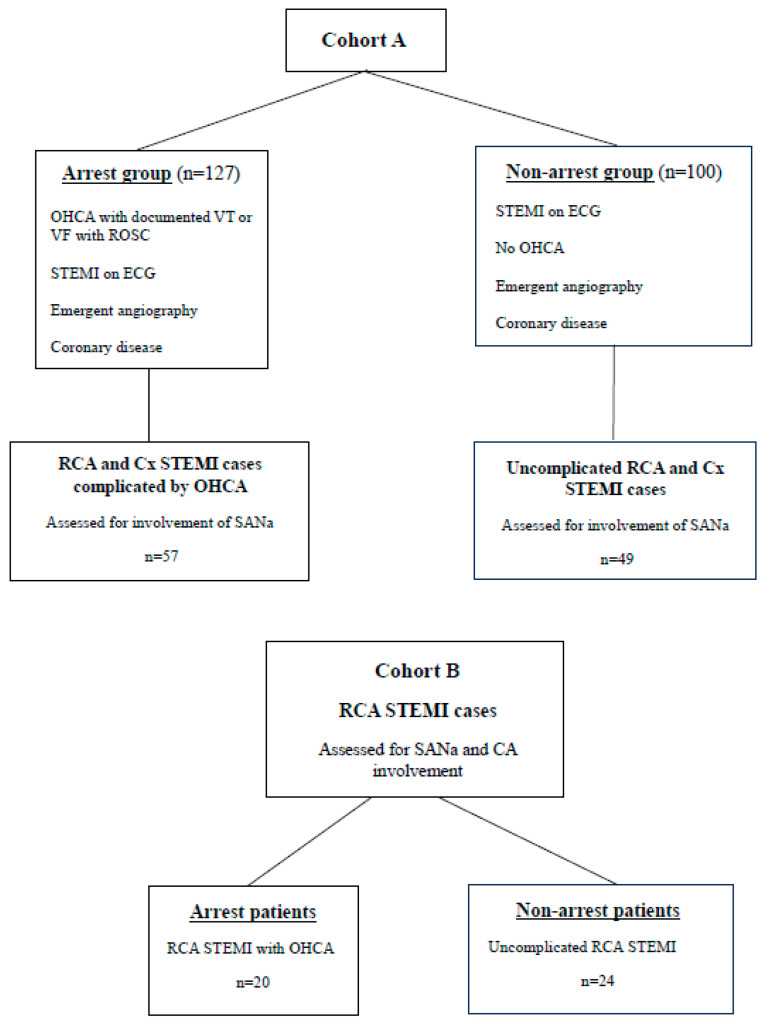
Study populations. Cohort A comprised 127 STEMI patients in the arrest group and 100 STEMI patients in the non-arrest group. Inclusion criteria as listed. Patients with RCA and Cx STEMIs in both these groups were assessed for SANa involvement. Cohort B: A separate population of patients with RCA STEMIs, 20 presenting with OHCA and 24 presenting with uncomplicated STEMI. This population was studied for SANa and CA involvement. OHCA = out-of-hospital cardiac arrest, VT = ventricular tachycardia, VF = ventricular fibrillation, STEMI = ST-elevation myocardial infarction, RCA = right coronary artery, Cx = circumflex artery, SANa =sinoatrial nodal artery, CA = conus artery.

This was a retrospective, single-centre, case–control study. A total of 127 consecutive STEMI patients (arrest group) with aborted SCD due to ventricular tachycardia and/or ventricular fibrillation (VT/VF) presenting from 2010 to 2014 inclusive were compared with 100 patients (non-arrest group) with uncomplicated STEMI admitted over the same time period. These patients were designated as Cohort A (Figure 1).

All cardiac arrest patients had documented pulseless VT or VF prior to successful resuscitation and hospital admission. Emergent angiography was performed on all patients in Cohort A as per the local primary PCI protocol and all had coronary pathology. Data was collected from medical notes, local database entries, procedural reports, 12 lead ECGs and angiographic reports and images.

ECG diagnosis of STEMI was defined as ST-segment elevation of 0.1 mV or more in any 2 contiguous leads, apart from 0.2 mV or more in men in leads V2 and V3, or new LBBB with a presentation consistent with acute coronary syndrome. Further ECG analysis was conducted by a study investigator, blinded to presentation, to assess the presence of lead V1 and V2 ST-segment shift. ST elevation in these leads is associated with RVOT ischaemia [7,8,9].

A SYNTAX score and Modified Jeopardy score (MJS), from invasive coronary angiography acquired from orthogonal views, were analysed as previously described [10,11,12,13], and patients with RCA or Cx territory MI were also assessed for SANa involvement. Angiographic assessment was carried out by an investigator blinded to presentation. Patients received standard, guideline-recommended AMI treatments including revascularization, medical and device therapy as necessary. Hospital and long-term (3.8 to 8.7 years following the index event) follow-up data were collected for Cohort A, and cause of death documented from death certificates.

A separate cohort of patients with STEMI involving the RCA only, Cohort B, was selected (Figure 1), and comprised 20 patients with inferior STEMI, complicated by cardiac arrest due to documented VA, and 24 patients with uncomplicated inferior STEMI. In this group, only angiographic images were reviewed for involvement of the SANa or CA by an investigator blinded to presentation.

Continuous variables are presented as mean and standard deviation and were compared using Student’s *t*-test. Categorical variables are presented as percentages and compared with the chi-squared test. A *p*-value of ≤0.05 was considered significant. Cumulative survival is presented in a Kaplan–Meier plot.

## 3. Results

### 3.1. Clinical Characteristics

In Cohort A, age, gender distribution and the comorbidities of hypertension and diabetes mellitus were similar across both the arrest and non-arrest groups (Table 1).

In 81% of arrest patients, this was their first manifestation of CAD, with a small minority in each group having had prior coronary intervention (percutaneous intervention or bypass grafting). There was no significant difference in overall prior documented CAD between the arrest and non-arrest groups.

### 3.2. Electrocardiogram Findings

There was no significant difference in the heart rate on presentation between arrest and non-arrest groups. There was a numerically higher incidence of atrial arrhythmia and complete heart block in the arrest group, but this did not reach significance. In Cohort A, there was an almost a threefold higher incidence of ST elevation in leads V1-V2 in patients with RCA culprit lesions in the arrest group compared with the non-arrest group (62% vs. 23%, *p* < 0.005). In Cohort B (Table 2), V1-V2 ST elevation was seen in 85% of the arrest group and 21% of the non-arrest group (*p* < 0.005).

### 3.3. Coronary Angiogram Findings

**Figure 2:**
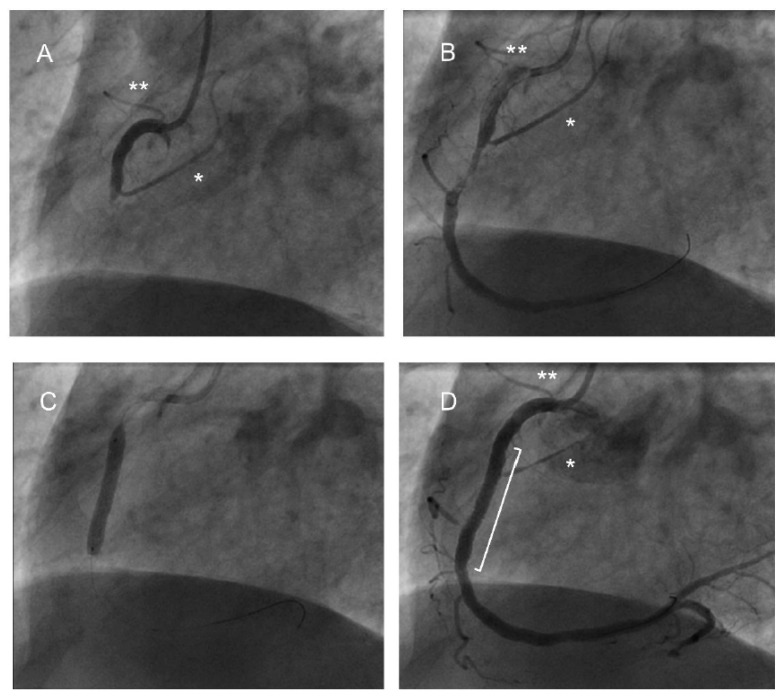
Angiogram images from a patient presenting post-VF arrest in the context of an inferior STEMI. The right coronary artery is shown in the RAO orientation. (**A**) Obstruction to flow at the mid-vessel level, just distal to the slightly pinched origin of the conus artery (*) with compromised flow within this branch. The sinoatrial nodal artery can be seen arising from the proximal RCA (**). (**B**) An angioplasty wire has been passed through to the distal RCA, restoring patency, and showing disrupted plaque in the mid-section. (**C**) Balloon deployment. (**D**) Post-stent deployment (stent margins encompassed by ]), with flow in the main vessel restored but further compromised flow in the conus artery, the origin of which is within the stent margins.

Single vessel disease was found in the majority, and with similar incidence, in both groups. There was almost equivalent incidence of both LAD and RCA/Cx culprit vessels in both the arrest (LAD 44%, RCA 34%, Cx 16% SVG/3VD 6%) and non-arrest (LMS 1%, LAD 38%, RCA 49%, Cx 10%, SVG 2%) groups (*p* = 0.09). More proximal lesions were identified in the arrest group but there were no differences in the SYNTAX or Modified Jeopardy scores between the groups. In Cohort A, patients with RCA/Cx culprit lesions had significantly more SANa involvement in the arrest group compared with the non-arrest group (77% vs. 33%, *p* < 0.005). In Cohort B (Table 2), 74% of patients in the arrest group had SANa or CA involvement, compared with 21% of the non-arrest group (*p* < 0.005). Procedural angiogram images from a patient with both the SANa and CA arising from the RCA are shown in Figure 2.

### 3.4. Cardiac Intervention

There were no significant differences between intervention performed on both groups in Cohort A (*p* = 0.72). In the arrest group, 92% underwent PCI, 3% underwent CABG, 1% had a device intervention (two patients: one had CRT-P upgrade to CRT-D and one patient had an ICD inserted) and 4% (five patients) had no intervention performed (one had coronary dissection treated conservatively, one had coronary spasm, one had a prior CABG and no defined targets, one had a non-dominant RCA occlusion which was not intervened upon and one had a chronic total occlusion of the RCA). Two patients had non-dominant RCA occlusion as the culprit. This compares with 93% of non-arrest patients treated by PCI, 2% with device interventions (one PPM for complete heart block and one PPM for sick sinus syndrome) and 4% (five patients) without any intervention (one had no targets defined, two had failed PCI, one had a distal PLV occlusion not suitable for intervention and one was a late presentation of an occluded LAD, and also later had hypertrophic obstructive cardiomyopathy).

### 3.5. In-Hospital Outcomes and Long Term Mortality Data

**Figure 3:**
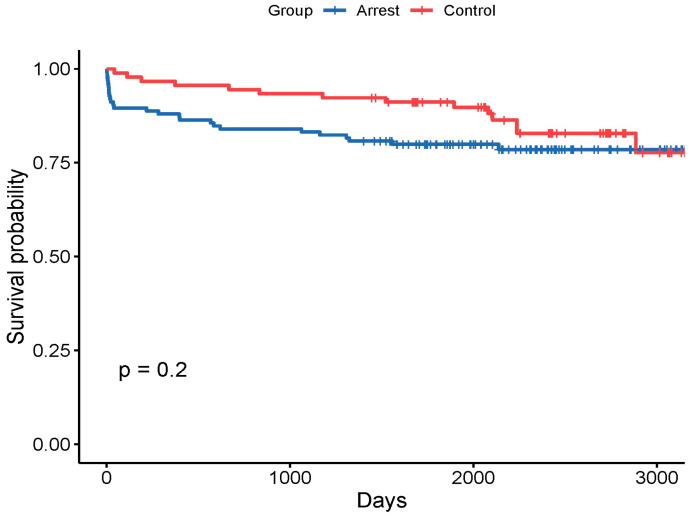
Kaplan–Meier curves with Landmark analysis, showing survival following index event of either acute myocardial infarction associated with cardiac arrest (arrest group), or uncomplicated acute myocardial infarction (control or non-arrest group).

There was a significantly higher 30-day mortality in the arrest group compared with the non-arrest group (43% vs. 7% of deaths, *p* < 0.05) but this difference was not sustained beyond 30 days (Figure 3). At the end of the follow-up period, the proportion of deceased patients (all-cause mortality) in the arrest group remained numerically higher, although not statistically significantly different (28 patients, 22% vs. 14 patients, 16%; *p* = 0.28), and occurred earlier.

The deceased in the arrest group had a higher mean SYNTAX (11 vs. 6, *p* < 0.005) compared with survivors, but no other angiographic parameters predicted long-term survival.

**Figure 4:**
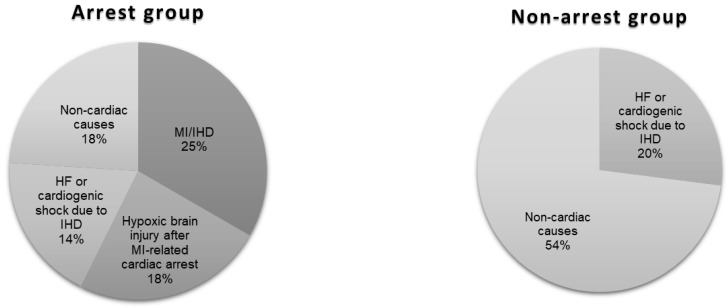
Causes of death for 75% of the arrest group and 74% of the non-arrest group (in patients where cause of death data was available). MI = myocardial infarction, IHD = ischaemic heart disease, HF = heart failure.

The majority of known causes of deaths in the arrest group were ascribed to cardiac pathology compared with the non-arrest group (57% vs. 20%, *p*-value <0.001) (Figure 4). Half of these deaths in the arrest group occurred within 30 days of the index event, compared with only 33% of total cardiac deaths in the non-arrest group (*p* = 0.015).

## 4. Discussion

The baseline characteristics of the patients in our study are similar to previously published data regarding aborted SCD secondary to acute coronary occlusion [14,15,16]. A male preponderance, older age, cardiac arrest as an initial manifestation of CAD, the high risk bestowed by hypertension, single vessel and proximal disease, and the presence of left coronary lesions have all featured in the previous literature.

There was preponderance of proximal LAD culprit lesions in the arrest group, likely explained by large anterior AMI being associated with high arrhythmic potential and worse outcomes. The extent and complexity of coronary disease and the amount of myocardium at risk, reflected in SYNTAX and Modified Jeopardy scores, were not significantly different between the groups. We noted that a significant number of arrests occurred in a non-LAD territory and, in particular, when the SANa was involved.

The LAD supplies a large territory of ventricular myocardium and is associated with larger myocardial infarctions and worse LVEF, both which promote VA and poorer prognosis [14,15,16]. However, RCA/Cx infarcts frequently contributed to our arrest group and have also been observed in a Danish study consisting of 219 patients with documented VF during their first STEMI prior to angioplasty, where 43% of patients had non-anterior infarcts [11]. Furthermore, the similar SYNTAX and MJS of both groups in Cohort A suggest that in our cohort, at least, the ischaemic burden and size of myocardium was not the dominant predictor of VA.

Sinus bradycardia is frequently associated with inferior AMI, particularly within the first few hours [17,18]. The Bezold–Jarisch reflex can be responsible—a triad of bradycardia, vasodilation and hypotension [19]—which is a cardioinhibitory reflex that can result from inferoposterior ischaemia [20]. There is some evidence to suggest that this reflex is involved in the bradycardia and hypotension encountered when the RCA is injected during coronary angiography [21].

Serrano et al. found a higher proportion of sinus bradycardia on admission in inferior AMI patients with proximal rather than mid or distal RCA occlusions [18]. Early experimental and clinical studies showed bradycardia was associated with VA in the context of myocardial ischaemia due to acute coronary occlusion [6,22,23]. It was proposed by Han et al. that either tachycardia or bradycardia could result in a dispersal of repolarisation and lead to VA [24].

In experiments performed on canine myocardium in vivo using epicardial and intramural needle electrodes, and simulating AMI by LAD ligation, Scherlag et al. found that small-amplitude, fractionated and delayed electrical activity was seen within infarcted epicardium and mid-myocardium, persisting beyond ventricular repolarisation [25]. During normal sinus rhythm, this fractionated activity resulted in local excitation and concealed re-entry, but under conditions of vagal-induced bradycardia, manifest re-entry causing closely coupled ventricular extrasystoles leading to VF, was observed.

We postulate that sinus bradycardia in the context of AMI may also result from ischaemia affecting the sinoatrial node. In our study, the SANa was compromised in the majority of non-LAD, particularly RCA, infarcts in the arrest groups. Although we did not demonstrate significant differences in heart rate between the arrest and non-arrest groups, possibly due to the administration of chronotropic drugs or partial recanalisation of the vessel, transient bradycardia at the beginning of the infarct process may be all that is required. Abrupt sinus bradycardia, coupled with hypotension provoking sympathetic upregulation, could potentially lead to enough repolarisation heterogeneity to trigger VA.

SANa occlusion resulting in transient bradycardia may be a potential mechanism for VA in the context of non-dominant RCA occlusion observed in a few case reports [4,5]. The SANa arises from the RCA in 60–70% of the population, and from the circumflex in about 20%, with an origin from the left main stem, LAD, aorta or bronchial artery in rare cases [26,27,28]. There is no definitive relationship between coronary dominance and the origin of the SANs, and two of the patients in our Cohort A arrest group had non-dominant RCA occlusion.

Another possible culprit for VA in the context of the RCA culprit AMI could be the conus artery (CA). The CA arises from the proximal RCA in 50–70% of cases and otherwise directly from the right sinus of Valsalva [29,30]. There have been several case reports citing chest pain, anterior ST elevation and recurrent VF associated with CA occlusion, either spontaneously, due to plaque shift or with stent occlusion [7,8,30,31,32], and coronary angiographers will be familiar with the risk of VF if the CA is injected. The CA supplies the RVOT, part of the anterior RV wall and the basal anterior interventricular septum. The RVOT has structural, cellular and electrophysiological properties that render it prone to arrhythmogenesis [33]. Connexin43 and SCN5A, both associated with rapid conduction, have reduced expression in the RVOT compared to the rest of the RV. There is both a wider range of action potential duration in this region, which can increase vulnerability to phase 2 re-entry, and altered calcium handling affecting sarcoplasmic reticulum that can result in triggered activity.

Separating the risk bestowed by the extent of myocardial ischaemia from that of specific coronary branch occlusion presents a challenge. Extent of at-risk myocardium can be a confounding factor when comparing proximal vs. mid/distal occlusions. Evidence to support the existence of variables beyond the burden of myocardial ischaemia would be the occurrence of cardiac arrest in the context of non-dominant RCA occlusion, as described earlier, and comparable coronary scoring between the arrest and non-arrest groups. We found no statistical difference in the SYNTAX and the Modified Jeopardy score between the two groups, suggesting it was not simply the amount of myocardium at risk which predicted the arrhythmic outcome.

There was a bimodal distribution of death in the arrest group, with the first peak within the first 30 days and the second between 1 and 5 years. In the non-arrest group, there was a unimodal distribution, with the highest proportion of deaths from 5 years beyond the index event to the end of follow up. This agrees with a finding in a similar study by Omer et al. [34], where the occurrence of cardiac arrest or cardiogenic shock increased short-term mortality significantly. Our study found that beyond 30 days, there was no significant difference in all-cause mortality between the groups, suggesting that the occurrence of VA at the index event does not affect long-term prognosis beyond 30 days.

An MI prior to the index event and higher SYNTAX in the deceased arrest group patients compared with survivors is in accordance with a higher burden of coronary disease or a further cardiovascular event conferring a poorer prognosis [35]. There was no such difference in the non-arrest group, bearing in mind that of most the causes of death known to us in this group were non-cardiac in nature.

## 5. Limitations

Our study was a small observational study that needs confirming in larger numbers of patients. It was underpowered to attribute survival to SANa or CA involvement due to our small cohort size and multiple factors involved in predicting mortality post MI. Cardiac arrest is possibly a predictor of large infarcts on the whole, and global ischaemia as a consequence of low flow during cardiopulmonary resuscitation would impact short-term survival. The cause of death was only available in three-quarters of the population, and was obtained from death certification completed by multiple clinicians at different sites, rather than being adjudicated.

## 6. Conclusions

A significant proportion of VA causing cardiac arrest in patients with AMI occur in non-LAD territories, suggesting more than simply infarct size being important in determining the risk of VA. Involvement of the SANa, resulting in bradycardia, and/or CA, resulting in ischaemia to the RVOT, may significantly contribute to the risk of VA. Transient disturbances of SANa and CA do not appear to translate into long-term adverse outcomes within the limits of our follow-up period.

## Figures and Tables

**Table 1:** Clinical characteristics of Cohort A study population with Cohort B ECG and angiography data included. Categorical variables represented as percentages. Continuous variables represented as mean (±standard deviation). Percentage deaths given as proportion of whole deceased population in group. NS = non-significant, CAD = coronary artery disease, bpm = beats per minute, STE = ST elevation, V_1_-V_2_ = standard 12-lead ECG leads V1 and V2, LMS = left main stem, LAD = left anterior descending artery, RCA = right coronary artery, Cx = circumflex artery, SVG = saphenous vein graft, 3VD = three-vessel disease, SANa = sinoatrial nodal artery, CA = conus artery.

Variables	Arrest Patients	Non-Arrest Patients	*p*-Value
Age	62 (±13)	62 (±14)	NS
Gender, male	76%	71%	NS
Hypertension	27%	34%	NS
Diabetes mellitus	13%	15%	NS
Prior documented CAD	19%	15%	NS
Electrocardiogram:			
Heart rate (bpm)	80 (±20)	77 (±15)	NS
Atrial arrhythmia	14%	9%	NS
Complete heart block	4%	2%	NS
STE > 1 mm V1-V2 (Cohort A: RCA culprits only)	62%	23%	<0.005
STE > 1 mm V1-V2 (Cohort B)	85%	21%	<0.005
Angiography:			
Single vessel disease	57%	60%	NS
Culprit vessel	LMS 0% LAD 44% RCA 34% Cx 16% SVG/3VD 6%	LMS 1% LAD 38% RCA 49% Cx 10% SVG 2%	NS
Proximal lesion	55%	41%	<0.05
SYNTAX score	14 (±10)	13 (±8)	NS
Modified Jeopardy score	6 (3)	6 (2)	NS
SANa involvement of RCA/Cx	77%	33%	<0.005
CA involvement of RCA (Cohort B)	74%	21%	<0.005
Index event intervention:			
No intervention	4%	5%	NS
Percutaneous coronary intervention	92%	93%	NS
Coronary artery bypass graft	3%	0%	NS
Device	1%	2%	NS
Long-term outcomes:			
Deceased	n = 28	n = 14	NS
Death ≤ 30 days	43%	7%	<0.05

**Table 2:** Cohort B study population: All patients had RCA infarcts, with 20 complicated by out of hospital cardiac arrest. STE = ST elevation, SANa = sinoatrial nodal artery, CA = conus artery.

	RCA Infarcts Complicated by Cardiac Arrest (n = 20)	Uncomplicated RCA Infarcts (n = 24)
STE > 1 mm V1-V2	85%	21%
SANa or CA involvement	74%	21%

## Data Availability

The data presented in this study are available on request from the corresponding author.
